# Supplementary material for: Red and blue language: Word choices in the Trump and Harris 2024 presidential debate
Source: PLoS One. 2025 Jun 3;20(6):e0324715. doi: 10.1371/journal.pone.0324715 (PMC12132968; doi:10.1371/journal.pone.0324715)
Supplement: Attachment — Submitted filename: Response to Reviewers.pdf [file pone.0324715.s001.pdf]

Dear Reviewers and Editor,

We sincerely appreciate your valuable and constructive feedback. We have carefully considered your comments and have integrated the necessary revisions into the main manuscript. For clarity, all modifications have been marked in red.

We have also been advised to remove the funding statement from the manuscript and will add it via Editorial Manager. Nonetheless, we've been instructed to include it in an updated cover letter, which we hereby do:

Marianna Bolognesi is partly funded by the European Research Council (ERC-2021-STG-101039777, project ABSTRACTION). Views and opinions expressed in the present paper are however those of the author(s) only and do not necessarily reflect those of the European Union or the European Research Council Executive Agency. Neither the European Union nor the granting authority can be held responsible for them. The funders had no role in study design, data collection and analysis, decision to publish, or preparation of the manuscript.

Thank you for your time and consideration.

Reviewer 1:

Reviewer #1: I like the general idea of the contribution. The corpus seems rather modest for the amount of analyses carried out, but the manuscript can be considered a case study that shows how such analyses can be done.

Response: Thank you. The size of the corpus is determined by the number of words uttered by the speakers during the debate. We agree that this can be considered a case study on this specific type of textual genre. We view our work as a focused case study and are encouraged by the potential for our methodology to be applied to future political debates, fostering further comparative research.

Reviewer #1: One of the most innovative aspects of the study is the use of the OpenAI LLM to identify the framing. While the inter-rater agreement of the human raters is discussed, it would be particularly relevant to know to what extent there is consistency in what the LLM does and the ratings. I understand that the raters worked on the framings identified by the LLM only (and did not themselves work through the corpus to identify the framings). It would still be interesting to get an idea of how many instances were considered 'falsely identified' by the raters.

Response: Thank you for emphasizing the importance of comparing the LLM's outputs with human ratings. We agree that this consistency metric is essential. In our online repository, we have reported detailed agreement statistics. Initially, we aimed to keep the manuscript's focus

on the application and investigation of framing identification rather than on a methodological deep dive. However, based on your feedback, we have now included these metrics in the manuscript, along with a clear reference to the repository for further details.

To elaborate, our analysis shows that in 62.10% of cases both annotators agreed that the framing identified by the LLM was correct. In an additional 28.66% of cases, one annotator agreed while the other did not, meaning that in 90.76% of instances the LLM's output was validated by at least one annotator - and 9.24% of framings noted falsely identified. We believe these findings highlight the robustness of the LLM in capturing relevant framing, even accounting for some of the inherent subjectivity in human judgment.

Reviewer #1: Another general comment would be that while the number of indices generated with automatic processing is impressive, it is not always very clear why these indices (termed "studies") are always relevant for the analysis of political discourse.

Response: Thank you for your comment. The indices we generated were not selected arbitrarily; rather, they were carefully designed to operationalize and quantify key variables outlined in our hypotheses. These indices allow us to systematically measure differences in political discourse between the two parties, providing a structured and replicable way to test our expectations. Without these metrics, the analysis would lack the necessary empirical foundation to assess variations in discourse systematically. By leveraging automated processing, we ensure consistency in measurement, reducing subjective biases while enhancing the robustness of our findings. We want to show two examples of the first two indices to demonstrate our line of argumentation in a more concise manner:

- Framing of Values and Ideology: "Metaphorical framing in political discourse plays a critical role in shaping how audiences perceive complex political issues. In a thorough meta-analysis [17] illustrate how metaphors frame topics and influence public attitudes and policy preferences. For instance …" which we connect to the identification of frames by providing a novel methodology of automatically identifying frames (Sec. 3.1). These identified frames are interpreted (Sec. 3.2.) in the context of the critical role they play in the debate - by discussing the identified frames quantitatively and qualitatively

- Appeals to Emotion and Logic: "Recent studies show that the use of emotionally engaging and empathetic language can impact politicians' appeal to voters by resonating with their personal experiences and values (e.g., [25])." Here, we need to identify and analyse the sentiment for both politicians in order to assess their appeal to the audience. We apply state-of-the-art sentiment analysis models in order to provide both a qualitative and quantitative interpretation of the measured sentiments (Sec. 4.2).

Reviewer #1: Even though in general I genuinely think that the case study is potentially interesting for scholars working on political discourse, I believe the authors should do a more thorough job explaining why the specific operationalizations are adequate (and not simply

feasible because there are tools on huggingface that can do something).
Thus I recommend major revisions.

Response: Thank you for your feedback. In this study, we deliberately adopted a top-down approach. We began by conducting a thorough review of the scientific literature on political discourse, with a particular focus on American political discourse. Based on this foundation, we formulated hypotheses regarding potential differences that may emerge in the specific context of the presidential debate between Trump and Harris last September.

To rigorously test these hypotheses, we developed a comprehensive framework for operationalizing the identified variables. Our approach incorporates a range of validated methodologies, including manual annotations, LLMs, and semantic norms, ensuring a robust and multifaceted analysis. Furthermore, we have substantiated the validity of our operationalizations by drawing on established scientific literature, which demonstrates that these metrics have been effectively employed across diverse contexts—ranging from discourse analysis to psycholinguistic experiments.

Given the wide range of available tools, we acknowledge that our selection may seem arbitrary, despite the fact that the need for these tools stems from a top-down approach. Other tools could potentially serve similar purposes. To address this, we will briefly outline additional technical reasons for our choices that were not included in the manuscript. We opted to prioritize the application and investigation aspects over more technical discussions:

- SpaCy lemmatizer: preferred over others because it is highly optimized for speed, integrates seamlessly with its robust NLP pipeline, and provides accurate, context-aware lemmatization with pre-trained linguistic models, unlike simpler rule-based alternatives.

- SiEBERT sentiment analysis: while there are many different language model-based approaches to sentiment analysis available, we decided to use SiEBERT, because is particularly effective for sentiment analysis in political debates due to its fine-tuning on diverse datasets, enabling it to capture the nuanced language and context-specific sentiments characteristic of political discourse. We also refer to works that use SiEBERT in the analysis of political discourse (Meijers, 2023) and speeched (Vazirani, 2023). In a pilot study, we have also tested alternative sentiment analysis models (TweetEval, focussing on tweet, hence inapt, BERTweet with the same issue) and realised that many available models are fine-tuned on twitter data for sentiment classification.

- spaCyTextBlob & Thesis Titan (fine-tuned mDeBERTa V3): as with the previous models, there is a vast variety of open-source models that can be used for subjectivity analysis. We provide the results of two models to ensure that our analysis is not biased by a single methodological approach. Comparing results from both models allows us to cross-validate our findings and increases the reliability of your subjectivity assessment.While spaCyTextBlob offers a well-established, rule-based method for estimating subjectivity, Thesis Titan (based on mDeBERTa V3) is a state-of-the-art

transformer model fine-tuned specifically for subjectivity detection. We believe that their complementary strengths help to cover different aspects of the subjectivity construct. Both models are grounded in a similar conceptual definition of subjectivity—the distinction between opinion-laden and factual statements—which aligns with our theoretical approach outlined in the respective section. Demonstrating consistency in theoretical assumptions across disparate models reinforces the validity of our approach. We want to utilize a rule-based approach (spaCyTextBlob) alongside a deep learning model (Thesis Titan) to provide a broader perspective on subjectivity. This mix helps us to ensure that potential limitations or biases in one model can be mitigated by the other.

- Political DEBATE (DeBERTa) Model: Our task was the identification of identity politics and group appeal. We have provided the theoretical discussion as to why we believe this study to contribute to the analysis. Now, in order to classify whether a statement belongs to the identity of one party over the other, we ideally fine-tune our own model on identity politics classification in order to avoid manual annotation and to provide an automated approach for this study. The political DEBATE model provides convincing results in relevant examples and our pilot studies to fulfil the need for a fine-tuned model for the exact purpose of this study - there is no other model available fine-tuned on political debates that can serve our purpose, unless we fine-tune our own.

We believe that our methodological approach ensures that our study is grounded in both theoretical and empirical precedent, reinforcing the reliability and relevance of our findings.

Reviewer #1: l. 396 “Recognizing the possibility of sentence dependence within responses, we acknowledge that some interdependence may exist between sentences, but argue that this dependence does not diminish the value of the statistical test, as political rhetoric often varies greatly sentence by sentence, making sentence-level analysis appropriate for capturing these shifts.”
The last statement is not supported by any citations or examples. I find it difficult to understand what exactly is meant, it looks a bit as if the problem of lack of independence of the sentences is explained away rather superficially. Please provide some more substantial argument here.

Response: Political speeches and written rhetoric are often deliberately designed with varied sentence-level constructions. Politicians may use one sentence to assert an opinion followed by another that presents a fact, or even interrupt a narrative to introduce a contrasting perspective. In such texts, the meaning and tone can change rapidly from sentence to sentence. This variability is consistent with findings in discourse analysis where authors use shifting rhetorical strategies to engage different audiences or to emphasize contrasting points (Gabrielsen, 2020 & Isaksen, 2011). An example of this can be seen in the following statement by Trump: "But they still bring it up just like they bring 2025 up. They bring all of this stuff up. I ask you this. You talk about the Capitol. Why are we allowing these millions of people to come through on the southern border?" In this statement, Trump shifts from referencing the January 6 insurrection ("they still bring it up") to Project 2025 ("like they bring 2025 up"), then to a more general

criticism of his critics ("They bring all of this stuff up"), and finally to a concern over border security ("people to come through on the southern border").

We have included this explanation and additional argumentation along with the example in the manuscript and hope it addresses the reviewers concerns about superficiality of the argument.

Reviewer #1: 559 “By maintaining a balance between presenting factual information and personal reflections, Harris’ objective responses are grounded in verifiable claims, while the subjective ones include more emotive, value-laden statements.”
This concluding statement is rather trivial and tautological: it is not a result but the author’s not really surprising assumption and operationalization that subjective statements are more value-laden.

Response: Thank you. Indeed, we removed the sentence, which we also considered to mostly contain redundant information.

Reviewer #1: 565 Not everybody know the construct of depth in the lexical hierarchy as it is implemented in WordNet. Please give some explanation what this refers to, and some examples. Is it related to lexical sophistication (which would be a more established construct in vocabulary research).

Response: Thank you for your suggestion. We appreciate the opportunity to clarify this point.

In WordNet, depth in the lexical hierarchy refers to the number of hierarchical steps (or levels) between a given word and the root of its semantic category. This depth represents how specific or general a word is within the hierarchy. For example, in WordNet’s noun hierarchy, "animal" is at a shallower depth than "dog," which in turn is shallower than "Golden Retriever." The deeper a word is in the hierarchy, the more specific its meaning.

While lexical depth and lexical sophistication are related, they are distinct constructs. Lexical sophistication generally refers to the use of less frequent, or more specialized vocabulary, whereas depth in WordNet’s hierarchy captures a word’s position within a structured semantic network. While deeper words can sometimes be more sophisticated, this is not necessarily always the case.

We added a short explanatory paragraph in the manuscript.

Reviewer #1: In this context, the authors argue that they focus on content words as an operationalization of Details vs. Big Picture and “filter out function words such as prepositions and conjunctions. This decision ensures that the analysis emphasises semantically rich vocabulary, thereby enhancing the robustness of our findings.”
This is not very convincing, as complexification also via conjunctions arguably allows speakers to provide a more nuance discussion than simply chaining main clauses. One has the impression that the feasibility and ease of obtaining scores from automatic algorithms trumps

(pun intended) a thorough operationalization of the target constructs.
The conclusion (602) that both candidates show comparable levels of **lexical** specificity is certainly empirically correct, but it should not be confounded with complexity in terms of their statements, for the aforementioned reasons.

Response: Thank you for your thoughtful feedback. We acknowledge the distinction between morphosyntactic and lexical complexity and clarify that our focus is on **semantic (lexical) complexity** (by which we mean the level of specificity, and therefore high-resolution semantics) rather than syntactic structuring. Research on text readability and comprehensibility (e.g., Collins-Thompson, 2014) demonstrates that word content plays a crucial role in determining text complexity, making it a robust measure for our analysis. Assessing morphosyntactic complexity in spoken dialogue presents significant challenges due to fluid clause boundaries and the spontaneous nature of speech. While morphosyntactic complexity is indeed a valuable aspect of linguistic analysis, it is more reliably applied to written texts, where sentence structures are more clearly delineated. Exploring this dimension would be an interesting avenue for future research but is beyond the scope of our current study, which prioritizes lexical complexity as the most appropriate metric for our hypotheses.

Reviewer #1: 614 Complexity of the language is operationalized via word concreteness. No rationale is given for this rather surprising choice, which is problematic given the huge amount of literature on complexity in first and second language production (cf. e.g. work by Biber, Housen, etc. etc.). Moreover, I do not understand why directness and complexity are treated as trade-offs, they seem rather orthogonal. Please explain why this should be the case, and provide relevant citations.

Response: Thank you for your comment. We clarify that we do not equate **complexity** with **concreteness** but rather use concreteness as a proxy for **clarity**. Words referring to concrete, tangible concepts tend to enhance clarity, as they facilitate shared understanding between speakers. In contrast, abstract words are more cognitively demanding to process due to their lack of direct referents. This distinction is well-supported in the scientific literature, including research on the **concreteness effect** (e.g., Jessen, 2000) and more recent behavioral studies on dialogue processing (e.g., Mazzuca et al., 2024). Furthermore, this idea aligns with classic discussions on language clarity, such as Orwell's recommendations in *Politics and the English Language*.

Reviewer #1: 763 “Similarly, Harris' two responses labelled as Republican also reflect a degree of crossover.” Again, this is a tautological ‘conclusion’: The very operationalization of the authors is that whenever a statement fits in with the opposite party's ideology, it is by definition crossover.

Response: Thank you for your comment. We would like to clarify that we explicitly acknowledge that this finding goes in the opposite direction of our initial prediction, which is why its interpretation must account for this unexpected result. In our discussion, we propose the "crossover" argument as a potential explanation for this pattern. However, we recognize that this

is just one possible interpretation, and we would be very interested in hearing any alternative explanations you might suggest. We appreciate potential reviewer's insights and look forward to any further thoughts they may have on this matter.

Reviewer #1: 859 "We argued that the more concrete the words are, the clearer the topic is addressed." I come back to the problematic use of lexico-semantic concreteness: In the conclusions, the authors now associate concreteness with clarity, while in the actual study concreteness was a sign of simplicity. Simplicity and clarity are not the same, an utterance may be complex but still clear; if the content matter is complex and abstract, it may be impossible to make it concrete while it may still be possible to remain clear. The analytical dimension complexity, clarity, and concreteness need to be better defined and more solid rationales need to be provided why and how they are related.

Response: Thank you for this insightful comment. We appreciate the opportunity to clarify our argument regarding the relationship between concreteness, clarity, and simplicity. We do not intend to conflate clarity with simplicity, nor do we suggest that all clear statements must be concrete. Rather, our argument is that increased lexical concreteness can contribute to clarity, particularly by reducing ambiguity and grounding discourse in tangible references. While it is true that complex ideas can still be conveyed clearly without being concrete, a substantial body of scientific literature supports the idea that conceptual concreteness is a valuable variable for operationalizing clarity. Studies on metaphor usage (e.g., Lakoff and Johnson 1980) demonstrate how concrete referents are frequently employed to enhance the understanding of (and therefore clarify) abstract concepts, while psycholinguistic research has consistently shown that concrete words are easier to process, understand, recall, produce, and memorize, compared to abstract concepts with the "concreteness effect" (Jessen et al., 2000).

Reviewer 2:

Reviewer #2: I enjoyed reading this manuscript, which is well-written and has clearly stated hypotheses to be tested. The hypotheses are well-grounded in previous literature underlying language use in speakers of different political parties. However, I do have some concerns related to the narrowness of the paper's scope and the lack of synthesis across the measures. Specific comments are given below.

Response: Thank you for your thoughtful and constructive feedback. We appreciate your positive remarks regarding the clarity of our writing and the grounding of our hypotheses in previous literature. We understand your concerns regarding the narrow scope of the paper and our measures. In response, we have addressed your specific comments below to strengthen our paper in accordance with your feedback.

Reviewer #2: Theoretical Development and Literature Review:

- The previous research that is cited is overly specific to differences in speech between political parties. While that is necessary, the paper would benefit from connecting more broadly to literature beyond political speech. For example, there is a wealth of literature on psychosocial constructs that underlie word choices (e.g., see James Pennebaker's work) or on linguistic relativity (e.g., see Lera Boroditsky's work; note also a paper by Chkhaidze et al., 2021, that specifically tests the influence of linguistic metaphors on attitudes toward immigration).

Response: We appreciate your insightful feedback. In the revised manuscript we have broadened our literature review beyond political speech differences. In addition to the politically focused studies, we now also discuss foundational work on psychosocial constructs underlying word choices. For example, we reference James Pennebaker's extensive research—which demonstrates that subtle aspects of language use (such as function word and pronoun usage) serve as reliable indicators of underlying psychological states and social dynamics (e.g., Pennebaker & King, 1999; Pennebaker et al., 2003). This literature not only informs our analysis of political discourse but also provides a broader framework for understanding the cognitive and affective processes reflected in language.

Moreover, we have integrated research from the field of linguistic relativity. Lera Boroditsky's work, for instance, compellingly shows that the structure of language can shape thought and perception, thus offering an important complement to our discussion (see Boroditsky, 2011). In line with this, we now also include the recent experimental study by Chkhaidze et al. (2021), which directly tests how subtle linguistic metaphors—such as those framing immigration—can systematically influence attitudes. We believe that by incorporating these broader streams of research, our paper not only gains in theoretical richness but also better situates our findings within a general framework of how language both reflects and shapes cognition and emotion.

Reviewer #2: While the authors do a good job of reviewing relevant literature for each aspect, what is missing for me is cohesion in bringing these particular measures together. Why were these particular measures selected, of the many that could have been analyzed? Is there any theory that ties them together?

Response: Thank you for this thoughtful comment. To the best of our knowledge, there is no cohesive theory that integrates all these aspects into a unified communicative style. This suggests that such a theory may still need to be developed.

In our case, our approach was driven by the goal of analyzing speech profiles based on specific hypotheses related to these variables—variables in which we expected to observe meaningful differences. While we acknowledge that other measures could have been considered, we selected these particular ones because they align with our research questions and the theoretical and empirical foundations available in the literature. We appreciate the reviewer's perspective and recognize the value of further theoretical development in this area.

Reviewer #2: Another reference to consider for broader perspective on natural language processing: Berger, J., & Packard, G. (2022). Using natural language processing to understand

people and culture. American Psychologist, 77(4), 525–537.
https://doi.org/10.1037/amp0000882

Response: Thank you! This is indeed a very apt reference and we have included it in our manuscript.

Reviewer #2: Results and Analyses: Again, in thinking about broader applicability, it would be interesting to compare measures in the present paper using Linguistic Inquiry and Word Count (LIWC-22) software, which counts words in psychologically meaningful categories. While not as sophisticated as the algorithms and models used to analyze the results in this paper, LIWC has been extensively used for text analysis in many papers and therefore could be useful in comparing whether some of the present results do or do not replicate. LIWC has measures of pronouns, positive and negative emotion words, and positive and negative tone, which could be comparable to similar measures reported in this paper. LIWC also includes measures of other psychological processes that could assess some of the hypothesized differences between Harris and Trump (e.g., drives such as achievement and power, or motives such as reward and risk).

Response: Thank you for your thoughtful suggestion. We agree that comparing our measures with those from LIWC-22 could be a valuable direction for future research. However, our methodology is designed to be open-source and cost-free - therefore reproducible in the context of other political debates (notably, as of today, GPT-4 via ChatGPT can be accessed for free and other large language models can be self-hosted). Since we do not have access to LIWC-22, integrating it into our methodology is not feasible - it is not open-source, not cost-free and there is no alternative. Moreover, our research follows a distinct analytical approach that is not directly aligned with LIWC-22's framework or our primary research hypotheses. Therefore, while an LIWC-22 comparison could be explored in future work, it falls outside the scope of our current study.

Reviewer #2: There is a lot to keep track of in this paper, so it would be helpful to have a table that shows each measure, the hypothesis for it, and the result.

Response: We believe that the structure of the paper aligns with the organization requested by the reviewer. Each variable of interest is introduced in the Theoretical Background (Sections 2.1 to 2.5) and is then individually examined in a dedicated study, clearly numbered (Study 1, Study 2, etc.). Within each study, we follow the standard research structure: methods, analyses, and discussion of results. Additionally, the main findings are summarized in the abstract. We have added the requested table on page 6, right before the studies are presented.

Reviewer #2: The authors should think about which figures are most critical for presenting the results (which ones are necessary to display the data from testing each of the hypotheses) and whether a subset of the data would be better presented in tables or in text.

Response: We appreciate the reviewer's input on our figure presentation. We took this suggestion seriously and carefully re-examined the figures to determine if any elements might be redundant or insufficiently detailed. However, without specific guidance on which aspects could be improved, and in light of the fact that this concern could not be unified with pointers by the other reviewer, we were unable to identify any particular issues with our current approach. Our decision to use this set of figures is based on their effectiveness in illustrating the nuanced trends and multifaceted interactions inherent in our data. We remain open to refining specific elements if further clarification is provided, but as it stands, we believe our presentation best communicates the results of our hypothesis testing.

Reviewer #2: Discussion: While the manuscript interprets each study's results independently, I didn't have a clear sense of the cumulative conclusions with respect to the central research question: "Are speech profiles of Trump and Harris systematically aligned with the typical characteristics identified in the literature as peculiar to Democrats and Republicans?" Not all of the measures demonstrated the expected patterns, so what is the overall conclusion? Are there measures that are given more weight than others when assessing the answer to this question?

Response: Thank you for this insightful comment. We acknowledge the need for a clearer synthesis of our findings in relation to the central research question. While some measures aligned with the expected partisan speech profiles, others did not, highlighting the complexity of political discourse. In the revised manuscript, we have added a summary paragraph in the Discussion section to explicitly address the overall conclusions and the relative weight of different measures in answering our main research question.

Reviewer #2: What is the contribution of this research beyond describing the speech profiles of two people? Some discussion of how the results extend previous research and tell us something new would be useful in documenting the research's contribution to the literature.

Response: Thank you for this important comment. Beyond describing the speech profiles of Trump and Harris in the debate held on 10 September, our study contributes to the literature by providing empirical evidence on how linguistic choices align with broader communicative strategies and political positioning. Our findings extend prior research on political discourse by showing how specific linguistic variables can differentiate (or not!) speakers, reflecting distinct rhetorical styles that resonate with different audiences. In fact, we would like to clarify that we have already addressed the connection between Harris and Trump's speech profiles and their respective political parties in the manuscript. Our analysis was not limited to describing their individual rhetorical styles but was explicitly framed in relation to broader partisan communication strategies. We discussed how their linguistic choices align with well-documented patterns in Democratic and Republican discourse.

Now that the election results are finalized (as our manuscript was written and submitted before election day), we need to make slight edits to ensure it does not sound anachronistic. This also presents an opportunity to reflect on how our findings relate to broader electoral outcomes. In

the revised manuscript, we will clarify these contributions to further strengthen the discussion on how our results inform the study of political communication.

References:

- Meijers, Yente. Investigating the Reciprocal Relationship between News and Parliament. A Study of Strikes in the UK Using Deep-learning Sentiment Analysis and Vector Autoregression. Lund University, Faculty of Social Sciences Graduate School. Thesis. (2023)
- Vazirani, Krish. "Evaluating the economic disparities in the world: sentiment analysis on central bank speeches from third world and first world countries." International Journal of Information Technology 16.1 (2024): 69-76.
- Gabrielsen, Jonas, Heidi Jønch-Clausen, and Christina Pontoppidan. "Answering without answering: Shifting as an evasive rhetorical strategy." Journalism 21.9 (2020): 1355-1370.
- Isaksen, Judy L. "Obama's rhetorical shift: Insights for communication studies." Communication Studies 62.4 (2011): 456-471.
- Collins-Thompson, Kevyn. "Computational assessment of text readability: A survey of current and future research." ITL-International Journal of Applied Linguistics 165.2 (2014): 97-135.
- Jessen, Frank, et al. "The concreteness effect: Evidence for dual coding and context availability." Brain and language 74.1 (2000): 103-112.
- Mazzuca, Claudia, et al. "Abstract Sentences elicit more Uncertainty and Curiosity than Concrete Sentences." Proceedings of the Annual Meeting of the Cognitive Science Society. Vol. 46. (2024).
- Lakoff, George, and Mark Johnson. "The metaphorical structure of the human conceptual system." Cognitive science 4.2 (1980): 195-208.
