# Supplementary material for: Red and blue language: Word choices in the Trump and Harris 2024 presidential debate
Source: PLoS One. 2025 Jun 3;20(6):e0324715. doi: 10.1371/journal.pone.0324715 (PMC12132968; doi:10.1371/journal.pone.0324715)
Supplement: Attachment — Submitted filename: Response_to_Reviewers_auresp_2.pdf [file pone.0324715.s002.pdf]

Response to Reviewers

We thank the reviewers for their thoughtful and constructive comments. Their insights have been very helpful in improving the clarity and quality of the work. We sincerely appreciate the time and effort put into reviewing our submission, and we have carefully considered and addressed each of your suggestions in our revisions. Here below, we summarize our revisions.

Main Comments:

My remaining issue is regarding the manuscript's general discussion and conclusion section, which could benefit from more connection back to the literature raised in the introduction and theoretical background sections (there are virtually no citations in the discussion). It would be helpful for the reader to see a better integration between these sections and also help to ground some of the conclusions in previous research.

Relatedly, I would like the authors to more explicitly address limitations of their research as well as directions for future research that build on their findings. They give a couple of examples in specific studies (one limitation in the fifth study and one future research direction in the first study), but a broader perspective on these issues would help to address my previous review comment about missing some cohesion in bringing these measures together. I understand that no overarching theoretical framework exists, but the authors could find other ways to connect the different studies, such as a limitations/future research section. I also see ways that the data in different studies could be linked; for example, Trump naming himself among the top 5 named entries (Study 1, Figure 3) seems consistent with the data from the Group Appeal analysis where Trump used more individualistic language (Study 5). Having the authors reflect on findings across studies, wherever possible, would enhance the reader's understanding of the findings and their implications.

Response

We thank the reviewer for this thoughtful and constructive feedback. We have taken this recommendation seriously and have revised the General Discussion and Conclusion section to better reflect the theoretical and empirical literature introduced earlier in the manuscript. All revisions are marked in red.

Specifically, we now explicitly reference prior work related to lexical specificity metrics, political communication styles, and linguistic abstraction in order to more clearly ground our findings in existing scholarship.

We also acknowledge the importance of articulating the limitations of our study in a broader and more cohesive manner. In response, we have elaborated on the methodological limitations associated with using WordNet's depth hierarchy as a proxy for specificity, and we have pointed to recent work by the Abstraction group that utilizes Best-Worst Scaling methods as a promising direction for overcoming these limitations. This change not only addresses the methodological concerns raised but also connects our study to evolving lines of research in the field.

We have included new insights on future research directions, suggesting multiple paths forward, including investigating the dynamics of specificity at the sentence and discourse levels, and integrating context-sensitive models. We also take a step back to reflect on how patterns observed across different studies in the manuscript (e.g., Trump's tendency to self-reference aligning with his individualistic framing) may in fact be interrelated, and we highlight how future work might further explore these cross-study connections.

We appreciate the reviewer's encouragement to strengthen the cohesion of the manuscript and believe these additions greatly enhance the clarity and scholarly contribution of our work.

Minor Comments:

I would like to thank the authors for the revised text. It is now more accessible for readers who might not be familiar with some or all of the NLP tools used. I genuinely think this is a highly useful contribution to the field as the methodologies described can be used for other corpora and to some extent also for other languages.
Minor comments that I have after reading the paper once again thoroughly:
Is it really necessary to aggregate the scores, e.g. the sentiment labels, for whole turns in the debate? I see why you want to do it to understand the timeline, but wouldn't it be better to treat each sentence as a unit of analysis and fit a logistic model with the predictor Trump/Harris to test the differences? (I could and should have made that comment after reading the first version, that's why I explicitly do not ask for revisions; this is just a thought that came to my mind when reading it once again).

typos and formal details: p. 4: "In line with this, is ..." --> delete the comma

I do not believe that the decimals are necessary in means of word counts (e.g. p. 6 "M=173.79")
p. 9: "If case of" --> "In case of"

Response

Thank you for the feedback. While a sentence-level approach might offer greater granularity, we argue that aggregating sentiment scores at the response level is methodologically more robust given our research context. Candidate turns (responses) in a political debate are often designed as a coherent communicative act, where adjacent sentences are contextually interdependent; breaking them apart for separate analysis risks violating key logistic regression assumptions, notably the independence and constant variance of observations - as serial correlation is highly likely within a single response. Analyzing the entire response allows us to capture the more coherent purpose behind a candidate's rhetoric, rather than noise from fragmented sentence-level fluctuations that may not reflect intentional messaging. Although exploring sentence-level sentiment could provide supplementary insights, our focus on response-level analysis directly aligns with established discourse frameworks and traces the change of messaging over the course of the debate.

Nonetheless, we were intrigued by your comment and would like to share results which will be provided in the paper accompanying code, but not the paper itself for the reasons outlined above: We have taken the SiEBERT sentiment model polarity labels by Trump and Harris at the sentence level using logistic regression, where the binary sentiment (positive = 1, non-positive = 0) is predicted by the candidate variable (with Harris as the reference group). Our analysis produced a coefficient of –0.5915 for the Trump indicator (SE = 0.127, z = –4.66, $p < 0.001$), which indicates that sentences attributed to Trump are significantly less likely to express positive sentiment than those attributed to Harris. Although the model's overall explanatory power is modest (Pseudo R^2 = 0.01341), the likelihood ratio test is significant ($p \approx 3.07e-06$), supporting the reliability of this difference.

Thank you for pointing out typos - we have corrected them.
